# NSAID-Induced acute kidney injury risk in patients on renin-angiotensin system inhibitors and diuretics: nationwide cohort study

**DOI:** 10.1186/s40780-025-00485-8

**Published:** 2025-08-18

**Authors:** Yuki Kunitsu, Daiki Hira, Shunsaku Nakagawa, Masahiro Tsuda, Shin-ya Morita, Yosuke Yamamoto, Tomohiro Terada

**Affiliations:** 1 Department of Clinical Pharmacology and Therapeutics, Kyoto University Hospital, 54 Shogoin-Kawahara-cho, Sakyo-ku, Kyoto, 606-8507 Japan; 2 Department of Pharmacy, Shiga University of Medical Science Hospital, Seta Tsukinowa-cho, Otsu, Shiga, 520-2192 Japan; 3 Graduate School of Pharmaceutical Sciences, Kyoto University, 46-29 Yoshida-Shimo-Adachi-cho, Sakyo-ku, Kyoto, 606-8501 Japan; 4 Department of Healthcare Epidemiology, Graduate School of Medicine and Public Health, Kyoto University, Yoshidakonoe-Cho, Sakyo-Ku, Kyoto, 606-8501 Japan

**Keywords:** Acute kidney injury, Non-steroidal anti-inflammatory drugs, Renin-Angiotensin system inhibitors, Diuretics, Triple whammy, Case-crossover analysis

## Abstract

**Background:**

Triple Whammy (TW) therapy, a combination of renin-angiotensin system inhibitors (RASIs), diuretics, and non-steroidal anti-inflammatory drugs (NSAIDs), is associated with an increased risk of acute kidney injury (AKI). However, there is no consensus regarding the impact of NSAID type on the risk of AKI. Therefore, in this study, we evaluated the incidence and risk of NSAID-induced AKI in patients taking concomitant RASIs and diuretics, focusing on NSAID type.

**Methods:**

We conducted an observational retrospective cohort study using a Japanese medical claims database. In the cohort analysis, 41,904 patients who received concomitant RASIs, diuretics, and newly added NSAIDs between April 2020 and March 2021 were included to estimate AKI incidence. In the case-crossover analysis, 2,909 patients who developed AKI while on RASIs and diuretics were analyzed to assess the short-term risk associated with NSAID use. Incidence rates were calculated using the person-year method. Conditional logistic regression was used to estimate adjusted odds ratios (aOR), accounting for surgical procedures and concomitant AKI risk drugs.

**Results:**

Among 41,904 patients, 54 developed AKI (20.0 [95% CI: 14.8–25.6] per 1,000 person-years). The incidence rate ratio of TW to RASIs and diuretics without NSAIDs was 2.08 [95% CI: 1.58–2.74]. Case-crossover analysis showed an aOR of 1.44 [95% CI: 1.17–1.78] for AKI associated with NSAID use. No substantial differences were observed between COX-2 selective and nonselective NSAIDs (aOR: 0.99 [95% CI: 0.66–1.50]).

**Conclusions:**

The addition of NSAIDs to RASIs and diuretics significantly increased AKI risk, emphasizing the need for careful monitoring regardless of the NSAID type.

**Supplementary Information:**

The online version contains supplementary material available at 10.1186/s40780-025-00485-8.

## Background

A risk of acute kidney injury (AKI) has been reported with the concomitant use of renin-angiotensin system inhibitors (RASIs), diuretics, and non-steroidal anti-inflammatory drugs (NSAIDs) [1–4]. This three-drug combination is referred to as ‘Triple Whammy’ (TW). In our previous study, we found that 65% of TW cases were due to the addition of NSAIDs [5] and that the time to AKI onset was earlier in TW cases with the addition of NSAIDs, with a median of 6.5 days [6]. In contrast, Camin et al. evaluated the incidence rate of AKI during the period of TW and reported an 8.82 [95% Confidence Interval (CI): 4.4–17.3] (/1,000 person-year) incidence rate of AKI due to TW over a 15-month follow-up period [7]. However, TW was defined as the three-drug combination at the initiation, and the duration of concurrent use was not assessed. Whereas Lapi et al. compared the AKI risk due to TW according to whether the NSAIDs used had a half-life of more than 12 h, but no significant differences were reported [4].

Inhibition of prostaglandin production by NSAIDs, via cyclooxygenase (COX) inhibition, and prostacyclin synthesis leads to contracture of the afferent arteriole, which in turn reduces renal blood flow and glomerular filtration rate, leading to AKI [8]. NSAIDs can be classified as selective COX-2 inhibitors (sCOX2-i) or nonselective COX inhibitors (nsCOX-i) based on their mechanism of action. Celecoxib, an sCOX2-i, has a lower impact on blood pressure elevation and a reduced risk of renal events [9–11]. However, COX-2 expression has also been observed in the kidney [12], with some studies suggesting that sCOX2-i should be avoided in patients with CKD and congestive heart failure, similar to nsCOX-i. Thus, there is no consensus regarding the impact of COX-2 selectivity on the risk of AKI [13]. Therefore, in the present study, we investigated the incidence of AKI associated with TW involving the addition of NSAIDs and compared the risk of AKI according to the specific NSAIDs used, utilizing a large Japanese medical database.

## Methods

### Data sources

We used data from the DeSC database provided by DeSC Healthcare Inc. (Tokyo, Japan). The database contains medical claims data for approximately 12 million individuals across various Japanese insurance systems, with an age distribution representative of the Japanese population [14, 15].

### Study design

This retrospective cohort study included two analyses: Analyses 1, 2. Patients concurrently using any RASIs and diuretics between April 1, 2020, and March 31, 2021, were selected as the base cohort (the RD cohort) from the DeSC database. The index date was defined as either the start date of the study period (April 1, 2020) or the start date of the concomitant use of RASIs and diuretics. If the same patient had multiple concomitant periods of RASI and diuretic use, only the first period was included in the study. Patients were excluded if: they were younger than 20 years of age at the index date, were undergoing dialysis treatment, were diagnosed with AKI in the year before the index date, or had missing prescription or medical history data in the year before the index date. Additionally, patients were excluded if their observation period ended prior to the index date. A diagram of the RD cohort is shown in Supplemental Fig. 1.

### Analysis 1. Investigation of AKI incidence in TW with the addition of NSAIDs

Patients in the RD cohort who received NSAIDs during the study period while using both RASIs and diuretics were followed-up until one of the following: new AKI diagnosis, dialysis initiation, discontinuation of any of the three drugs, end of database observation, or 180 days after TW initiation. The AKI incidence was calculated using the person-year method. Patients who reached the endpoint on the day of TW initiation were excluded from the observation period because it was unclear whether the prescription or diagnosis occurred on the same day. Patients were also excluded if they had a history of AKI, dialysis, or NSAID prescriptions in the year before TW initiation or if they had only one day of TW therapy. We identified patients in the RD cohort who did not have NSAID prescriptions in the year before the index date and followed them until AKI diagnosis, dialysis, discontinuation of either drug, or NSAID initiation for comparison with the two-drug group (RASI and diuretics without NSAIDs). Subgroup analyses were conducted based on COX-2 selectivity and specific components of the added NSAIDs. In these analyses, follow-up was terminated upon switching to an NSAID with a different COX-2 selectivity or component.

### Analysis 2. Evaluation of the risk of AKI associated with COX-2 selectivity of NSAIDs in patients using RASIs and diuretics using a case-crossover approach

Patients the RD cohort who developed AKI were selected for the case-crossover analysis. NSAID use was compared between the case (1–30 days before AKI onset) and control periods (46–75 and 91–120 days before AKI onset). The case period was defined as the 1–30 days before AKI onset, based on previous pharmacoepidemiologic research indicating that the risk of NSAID-associated AKI is highest within the first month of treatment [4, 6, 16]. The control periods (46–75 and 91–120 days before AKI onset) were selected to provide appropriate washout intervals while preserving within-person comparability. The analysis was adjusted for surgical procedures and the use of other AKI-risk drugs. Only patients receiving continuous RASI and diuretic therapy for at least 120 days before AKI onset were included.

Each analysis was designed to address a distinct research question: Analysis 1 (incidence analysis) aimed to estimate the long-term incidence of AKI following TW initiation, while Analysis 2 (case-crossover) focused on evaluating the short-term risk of NSAID use immediately prior to AKI onset. This dual-approach design allowed for complementary insights into both the temporal pattern of AKI occurrence and the potential triggering effect of NSAIDs within the TW regimen.

### Exposure

RASIs, diuretics, and NSAIDs were defined according to the Anatomical Therapeutic Chemical (ATC) classification system (Supplemental Table 1). NSAIDs were classified as COX-2 selective or nonselective based on their pharmacologic profiles and regulatory labeling. A full list of included agents is presented in Supplemental Table 1. Topical drugs such as tapes and ointments were excluded. The duration of use of each drug was defined as the period from the date of dispensing to the number of days for which the drug was prescribed, excluding as-needed prescriptions. Given that patient adherence to medications and follow-up consultations may not have been perfect, a 30-day grace period was considered. Concomitant use of RASIs and diuretics was defined as overlapping periods of drug use, allowing for the grace period. The addition of NSAIDs during this overlapping period was considered TW exposure. For patients prescribed multiple agents within the same therapeutic class (i.e., RASIs, diuretics, or NSAIDs), drug use was considered persistent if at least one agent in the class remained active during the period of interest, including the grace period.

### Definition of outcome and covariates

The primary outcome of the study was the new diagnosis of AKI, identified using the International Classification of Diseases, 10th Edition (ICD-10) code N17X, based on previous studies which indicate that this code has moderate sensitivity and high specificity for identifying AKI [17]. The date of the act with the Japanese procedure codes J038 or C102 was defined as the date of dialysis. Patient background factors including surgical procedures, and medical histories (known AKI risk factors) were assessed. Surgical procedures were defined using the Japanese procedure codes K00–91 and K93. The respective ICD-10 diagnosis codes are provided in Supplemental Table 2 [18–26]. In addition, drugs associated with AKI risk, other than RASIs, diuretics, and NSAIDs, were defined based on the guideline [27] and a previous study [28] (Supplemental Table 3), and short-term fluctuations were considered by including antibiotics, antineoplastics, antivirals, corticosteroids, and iodinated contrast agents as covariates.

### Statistical analysis

In Analysis 1, AKI incidence rates were calculated using the person-year method, and 95% confidence intervals (CIs) were derived using Poisson distribution. In Analysis 2, conditional logistic regression was used to evaluate the association between NSAID use and AKI development, with drugs associated with AKI risk and surgical procedures as covariates. NSAID use during the case period (1–30 days before AKI onset) was compared with that during the control period (46–75 days and 91–120 days before AKI onset). To minimize the risk of overfitting in the multivariable conditional logistic regression models, crude odds ratios were calculated only when the event count exceeded 10. Adjusted odds ratios were estimated only when the total number of events was at least 10 times the number of covariates included, following the rule of thumb for logistic regression [29]. The significance level was set at *p* < 0.05. Python 3.115 was used to estimate confidence intervals for AKI incidence rate using the Poisson distribution, while other statistical analyses were conducted using JMP^®^ Pro 18 (SAS, Cary, NC, USA).

### Sensitivity analyses

Several sensitivity analyses were performed to assess the robustness of the results. The primary endpoints were redefined as AKI and dialysis, or AKI and acute interstitial nephritis (AIN). In this study, AKI was primarily defined as pre-renal AKI, which is commonly observed in TW therapy and is captured by the ICD-10 code [N17] [4]. However, NSAIDs can occasionally induce intrinsic renal AKI, which manifests as AIN and is defined by ICD-10 codes [N10] or [N14]. Therefore, AIN was included as a supplemental endpoint in the sensitivity analysis to account for potential variations in renal injury patterns and ensure robustness of the results. Additionally, the grace period for drug duration was adjusted to zero or seven days. Only patients with at least two dispensing events were considered NSAID users given that NSAIDs are often discontinued at the patient’s discretion based on pain severity. Furthermore, to address potential time-varying confounding, sensitivity analyses were conducted by additionally adjusting for calcineurin inhibitor use and intensive care unit (ICU) admission during the case and control periods. Calcineurin inhibitor use was defined based on prescriptions for drugs classified under the ATC code L04AD. ICU admission was identified based on the claim for the specific intensive care unit management fee. These factors were included based on their known association with AKI risk, even though their prevalence in the study population was low. Finally, in Analysis 2, the case and control periods were set at 14 days. The case period was defined as 1–14 days before AKI onset, whereas the control periods were defined as 31–44 and 61–74 days before AKI onset.

## Results

Overall, 463,694 patients in the dataset had taken RASIs and diuretic combinations for more than one day during the study period. After applying the exclusion criteria, a total of 365,649 patients were included in the RD cohort.

### Analysis 1. Investigation of AKI incidence in TW with the addition of NSAIDs

**Fig. 1:**
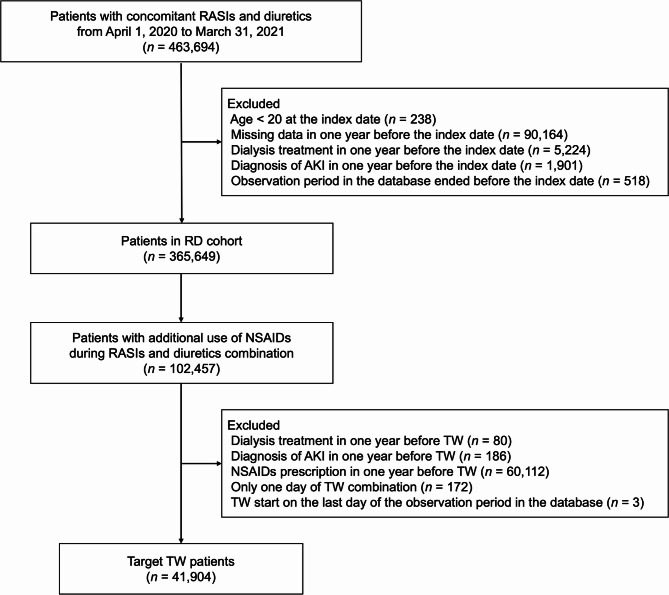
Flowchart of TW patient inclusion Abbreviations: AKI, acute kidney injury. NSAIDs, non-steroidal anti-inflammatory drugs. RASIs, renin-angiotensin system inhibitors. TW, Triple Whammy

In the RD cohort, 102,457 patients were identified as concurrently using RASI, diuretics, and NSAIDs. After applying the exclusion criteria, 41,904 patients were selected as the target group for TW with the addition of NSAIDs (Fig. 1). The background characteristics of the patients at the time of NSAID administration are presented in Table 1. During follow-up, 54 patients developed AKI. The AKI incidence rate in TW with added NSAIDs and in patients with RASI and diuretics without NSAIDs was 20.0 [14.8 − 25.6] per 1,000 person-years and 9.6 [9.0 − 10.0] per 1,000 person-years, respectively. The incidence rate ratio for the addition of NSAIDs compared to the two-drug combination of RASI and diuretics, was 2.08 [95% CI: 1.58–2.74]. The AKI incidence rates according to the type of NSAIDs added are shown in Table 2. AKI incidence rates according to baseline characteristics are shown in Supplemental Table 4.

**Table 1:** Baseline characteristics at the start of TW

Characteristics		
Male, n (%)	19,857	(47)
Age, years, median [range]	78	[22 − 106]
Follow-up duration, days, median [range]	7	[1 − 180]
Endpoints, n (%)		
AKI	54	(0.1)
Dialysis	14	(0.0)
End of any TW drugs	40,197	(96)
End of follow-up	150	(0.4)
180 days after TW start	1489	(4)
Medical history, n (%)		
Cancer	7656	(18)
Diabetes mellitus	22,006	(53)
Heart failure	22,266	(53)
Hypertension	41,623	(99)
Ischemic heart disease	15,160	(36)
AKI^a^	85	(0.2)
CKD	4873	(12)
Other renal disease	9958	(24)
Use of TW drugs, n (%)		
RASIs	41,904	(100)
ACEIs	4983	(12)
ARBs	37,567	(90)
Direct renin inhibitor	95	(0.2)
Diuretics	41,904	(100)
Loop diuretics	16,300	(39)
Potassium-sparing diuretics	8650	(21)
Thiazide diuretics	19,348	(46)
Thiazide-like diuretics	4130	(10)
Vasopressin V2 receptor antagonists	1227	(3)
NSAIDs	41,904	(100)
nsCOX-i	31,435	(75)
sCOX2-i	10,761	(26)

^a^The diagnosis of AKI within one year was an exclusion criterion

ACEIs, angiotensin-converting enzyme inhibitor. AKI, acute kidney injury. ARBs, angiotensin receptor blocker. CKD: chronic kidney disease. NSAIDs, non-steroidal anti-inflammatory drugs. nsCOX-i: nonselective cyclooxygenase inhibitor. RASIs and RAS inhibitors. sCOX2-i, cyclooxygenase-2 selective inhibitors. TW, Triple Whammy

**Table 2:** Incidence rate of AKI by type of added NSAIDs in TW patients

Added NSAIDs	*n*	AKI cases, *n*	Total follow-up duration, days	AKI incidence rate [95%CI] (/1,000 person-years)
**Any type**	41,904	54	984,848	20.0 [14.8 − 25.6]
**nsCOX-i**	30,968	32	493,273	23.7 [15.5 − 32.6]
Acemetacin	54	0	471	-
Aluminium flufenamate	19	0	124	-
Ampiroxicam	15	0	220	-
Diclofenac sodium	5495	5	44,931	40.6 [8.12 − 81.2]
Flurbiprofen	124	0	789	-
Flurbiprofen axetil	1443	3	2468	443.7 [0 − 1035.3]
Ibuprofen	262	0	2801	-
Indomethacin	88	0	292	-
Indomethacin Farnesyl	58	0	2272	-
Ketoprofen	328	0	2362	-
Loxoprofen sodium hydrate	21,870	18	381,522	17.2 [9.57 − 25.8]
Mefenamic acid	329	0	2166	-
Moffezolac	50	0	546	-
Naproxen	150	1	2995	121.9 [0 − 365.6]
Oxaprozin	6	0	90	-
Piroxicam	2	0	120	-
Planoprofen	187	0	1868	-
Proglumetacin maleate	8	0	364	-
Surindac	28	0	952	-
Thiaprofenic acid	32	0	200	-
**sCOX2-i**	10,425	15	383,861	14.3 [7.61 − 21.9]
Celecoxib	8367	12	321,744	13.6 [6.81 − 21.6]
Etodolac	746	1	22,677	16.1 [0 − 48.3]
Lornoxicam	565	0	9354	-
Meloxicam	296	1	13,376	27.3 [0 − 81.9]
Nabumetone	7	0	156	-
Zartoprofen	483	0	8742	-

Patients who were initiated on different types or components together were excluded from the analysis

The AKI incidence rate was calculated using the person-year method, dividing the number of AKI cases by the total follow-up period, and presented per 1,000 person-years

Abbreviations: AKI, acute kidney injury. CI, confidence interval. NSAIDs, non-steroidal anti-inflammatory drugs. nsCOX-i: nonselective cyclooxygenase inhibitor. sCOX2-i, cyclooxygenase-2 selective inhibitors. TW, Triple Whammy

### Analysis 2. Evaluation of AKI risk associated with COX-2 selectivity of NSAIDs in patients using RASIs and diuretics using a case-crossover approach

In the RD cohort, 3,706 patients developed AKI during the study period when using a combination of RASI and diuretics. Of these, 2,909 patients who had been on continuous RASI and diuretics for at least 120 days before the onset of AKI were included in the analysis. The background factors and concomitant medications administered during the case and control periods are shown in Table 3. Adjusted odds ratios for NSAID use by type or component during the case period, adjusted for concomitant use of other AKI risk drugs and surgical procedures, were calculated (Table 4). The adjusted odds ratios of nsCOX-i and sCOX2-i were 1.43 [95% CI: 1.13 − 1.80] and 1.42 [95% CI: 1.01 − 2.00], respectively. The relative odds ratio of sCOX2-i to nsCOX-i was 0.99 [95% CI: 0.66 − 1.50]. No significant differences were observed in the component comparisons.

**Table 3:** Characteristics of case and control period

Characteristics	case period (*n* = 2,909)		control period (*n* = 5,818)	
Male, n (%)	1532	(53)	3064	(53)
Age, years, median [range]	83	[23 − 104]	83	[23 − 104]
Surgery in the period, n (%)	172	(6)	162	(3)
Use of TW drugs in the period, n (%)				
RASIs	2909	(100)	5818	(100)
ACEIs	528	(18)	1064	(18)
ARBs	2451	(84)	4886	(84)
Direct renin inhibitor	4	(0.1)	11	(0.2)
Diuretics	2909	(100)	5818	(100)
Loop diuretics	2082	(72)	3961	(68)
Potassium-sparing diuretics	1089	(38)	1964	(34)
Thiazide diuretics	881	(30)	1740	(30)
Thiazide-like diuretics	166	(6)	316	(5)
Vasopressin V2 receptor antagonists	375	(13)	653	(11)
NSAIDs	501	(17)	804	(14)
nsCOX-i	322	(11)	467	(8)
sCOX2-i	212	(7)	371	(6)
Use of drugs associated with AKI risk, n (%)				
Antibiotics	723	(25)	752	(13)
Antineoplastics	83	(3)	149	(3)
Antivirals	35	(1)	44	(1)
Corticosteroid	341	(12)	572	(10)
Iodinated contrast agents	137	(4.7)	157	(2.7)

ACEIs, angiotensin-converting enzyme inhibitor. AKI, acute kidney injury. ARBs, angiotensin receptor blocker. NSAIDs, non-steroidal anti-inflammatory drugs. nsCOX-i: nonselective cyclooxygenase inhibitor. RASIs and RAS inhibitors. sCOX2-i, cyclooxygenase-2 selective inhibitors. TW, Triple Whammy

**Table 4:** Odds ratios for AKI by type or component of NSAIDs use during the case period

	case period (*n* = 2,909)		control period (*n* = 5,818)		Crude odds ratio [95%CI]	Adjusted^a^ odds ratio [95%CI]	*p*
any NSAIDs	501	(17)	804	(14)	1.98 [1.63 − 2.42]	1.44 [1.17 − 1.78]	< 0.001
any nsCOX-i	322	(11)	467	(8)	2.08 [1.68 − 2.59]	1.43 [1.13 − 1.80]	0.003
Acemetacin	1	(0.0)	0	(0)	-	-	-
Aluminium flufenamate	0	(0)	0	(0)	-	-	-
Ampiroxicam	0	(0)	0	(0)	-	-	-
Diclofenac sodium	79	(3)	84	(1)	2.71 [1.83 − 4.06]	1.79 [1.19 − 2.74]	0.005
Flurbiprofen	0	(0)	0	(0)	-	-	-
Flurbiprofen axetil	9	(0.3)	13	(0.2)	-	-	-
Ibuprofen	2	(0.1)	2	(0.0)	-	-	-
Indomethacin	1	(0.0)	4	(0.1)	-	-	-
Indomethacin Farnesyl	2	(0.1)	2	(0.0)	-	-	-
Ketoprofen	13	(0.4)	11	(0.2)	2.84 [1.17 − 7.29]	-	-
Loxoprofen sodium hydrate	228	(8)	350	(6)	1.94 [1.49 − 2.55]	1.43 [1.08 − 1.89]	0.01
Mefenamic acid	4	(0.1)	2	(0.0)	-	-	-
Moffezolac	0	(0)	0	(0)	-	-	-
Naproxen	2	(0.1)	3	(0.1)	-	-	-
Oxaprozin	0	(0)	0	(0)	-	-	-
Piroxicam	0	(0)	0	(0)	-	-	-
Planoprofen	3	(0.1)	5	(0.1)	-	-	-
Proglumetacin maleate	1	(0.0)	2	(0.0)	-	-	-
Surindac	0	(0)	0	(0)	-	-	-
Thiaprofenic acid	0	(0)	0	(0)	-	-	-
any sCOX2-i	212	(7)	371	(6)	1.66 [1.19 − 2.32]	1.42 [1.01 − 2.00]	0.05
Celecoxib	184	(6)	318	(5)	1.76 [1.23 − 2.52]	1.54 [1.06 − 2.23]	0.02
Etodolac	12	(0.4)	22	(0.4)	1.31 [0.36 − 4.48]	-	-
Lornoxicam	1	(0.0)	2	(0.0)	-	-	-
Meloxicam	13	(0.4)	27	(0.5)	0.85 [0.20 − 3.43]	-	-
Nabumetone	0	(0)	0	(0)	-	-	-
Zartoprofen	5	(0.2)	6	(0.1)	-	-	-

^a^adjustment factors: surgical procedures and use of antibiotics, iodinated contrast agents, corticosteroids, antineoplastic agents, antiviral drugs

Crude odds ratios were calculated only for items with event counts greater than 10. Adjusted odds ratios were calculated only for items with an event count greater than 60

Abbreviations: AKI, acute kidney injury. CI, confidence interval. NSAIDs, non-steroidal anti-inflammatory drugs. nsCOX-i: nonselective cyclooxygenase inhibitor. sCOX2-i, cyclooxygenase-2 selective inhibitors

### Sensitivity analysis

The results of the sensitivity analyses are presented in Supplemental Tables 5 and 6. Changing the primary endpoint increased the number of patients with AKI. The AKI incidence rate for the addition of NSAIDs in patients receiving RASIs and diuretics tended to be slightly lower than that in the main analysis when dialysis was included as the primary endpoint, however, no significant changes were observed.

Additionally, when the grace period was reduced, the number of patients included in the analysis decreased due to the shorter allowable period for concurrent use. However, some of the outcomes were not significant. Similarly, when only patients with at least two dispensing events for NSAIDs were included, the number of patients decreased, leading to some results losing significance.

Furthermore, when ICU admission and calcineurin inhibitor use were added as covariates in the case-crossover analysis, the results remained robust. ICU admissions were observed in 22 (0.8%) of the case periods and 14 (0.2%) of the control periods, while calcineurin inhibitor use was found in 25 (0.9%) and 46 (0.8%) of the respective periods. Despite their low prevalence, adjusting for these factors did not substantially alter the effect estimates.

The number of NSAID users during case and control periods decreased in Analysis 2, wherein they were shortened to 14 days. However, the relative adjusted odds ratio of sCOX2-i to nsCOX-i was consistent with that of the main analysis.

## Discussion

To the best of our knowledge, this study is the first to report AKI incidence during TW persistence and to classify risk based on NSAID composition and COX-2 selectivity. The median TW duration was 7 days, with an AKI incidence rate ratio of 2.08 [95% CI: 1.58 − 2.74] compared to the RASI plus diuretic group. Case-crossover analysis showed an adjusted odds ratio of 1.44 [95% CI: 1.17 − 1.78] for NSAID use. However, no significant differences were found between the NSAIDs.

Although previous studies have assessed the risk of AKI in patients with TW, its incidence rates remain unclear. Camin et al. reported an AKI incidence rate of 8.82 [95%CI: 4.4 − 17.3] (/ 1,000 person-years) in TW [7]. However, the definition of TW in this report was patients on three concomitant drugs at the time of initiation, and the continuity of TW was unknown. Whereas our study, which accounted for TW continuity, found a higher rate of 20.0 [95%CI: 14.8 − 25.6] (1,000/person-years). This discrepancy may be due to differences in follow-up duration and TW continuity. Additionally, in the present study, we used case-crossover comparisons. However, previous nested case-control studies reported increased AKI risk with TW (adjusted risk ratios: 1.31–1.64) [4, 30, 31]. Nested case-control analysis and case-crossover comparisons are used as risk analysis methods for rare adverse events such as AKI, which allow adjustment for background factors [32, 33]. Nested case-control analysis is considered suitable for the assessment of long-term risk factors, whereas case-crossover comparisons are suitable for the assessment of short-term factors [34–36]. Lapi et al. found that the highest risk of developing AKI with TW was observed within the first 30 days of NSAID use [4]. Similarly, in our previous study on the pattern of TW with the addition of NSAIDs, the time to AKI onset was short, with a median of 6.5 days [6]. Consistent with this, the results of Analysis 1 show that the median follow-up time to AKI onset was 7 days. Therefore, we opted for case-crossover comparisons for risk comparisons. Consequently, the adjusted odds ratio of NSAIDs use was significantly higher in patients with RASI plus diuretics, at 1.44 [95%CI: 1.17 − 1.78] in the immediate period prior to AKI onset. This is close to the TW-adjusted risk ratio and adjusted odds ratio in previous reports [4, 30, 31] and supports an increased risk of AKI with TW. Although the incidence analysis and the case-crossover analysis both evaluated the association between TW therapy and AKI, they were designed to address different clinical questions. The incidence analysis assessed long-term AKI incidence following TW initiation, whereas the case-crossover approach focused on the short-term risk immediately preceding AKI onset. As a result, the follow-up durations differed substantially (up to 180 days vs. 30 days, respectively), and the findings should not be directly compared. In the case-crossover analysis, we deliberately selected a 30-day case period based on previous studies suggesting that the risk of NSAID-induced AKI is highest within the first month of exposure [4, 6, 16]. Longer windows such as 60–90 days were not adopted to minimize the risk of time-varying confounding and exposure misclassification, thereby ensuring more accurate assessment of short-term drug effects. This highlights the need to interpret the observed short-term risk in a clinically meaningful context. This result suggests a clinically meaningful increase in AKI risk among patients already receiving RASIs and diuretics. Given the widespread use of these medications, even a moderate relative increase in risk may translate into a substantial number of preventable AKI cases, especially in elderly patients with reduced renal reserve. This highlights the importance of carefully assessing renal function and limiting unnecessary NSAID prescriptions in this vulnerable population. However, whether the observed increase in AKI risk is primarily due to the intrinsic nephrotoxic effects of NSAIDs or their interactions with RASIs and diuretics remains unclear. Previous studies have reported no baseline AKI risk across different populations, making absolute risk estimation challenging. However, baseline AKI risk, and consequently the absolute risk associated with NSAIDs exposure, is suggested to be higher in individuals with CKD and older adults [37]. Additionally, an increased risk of AKI has been demonstrated in patients with volume depletion [38]. Thus, the AKI risk associated with NSAID use has been shown to vary significantly depending on underlying conditions and concomitant medications [30]. Further research is required to identify the independent and interactive effects of NSAIDs within TW therapy.

The present results showed that 28% patients in the RD cohort used NSAIDs concomitantly with RASIs and diuretics, a rate approximately three times higher than the 8.8% reported in a UK study of older adults [39]. This suggests a higher prevalence of NSAIDs co-prescription in Japan, possibly influenced by the frequent use of NSAIDs [40, 41] and the aging population with orthopedic conditions [42]. Given the median age of 78 years in our cohort, the addition of NSAIDs requires careful consideration of age and concomitant medications to minimize AKI risk.

In Analysis 2, after adjusting for short-term AKI-related factors such as AKI risk drugs and surgical procedures, no significant difference was observed between sCOX2-i and nsCOX-i (adjusted relative odds ratio: 0.99 [95% CI: 0.66–1.50]). Furthermore, no NSAID showed significant risk variations when analyzed by component. Given that this analysis accounted for these short-term risk factors, it provided a more reliable risk assessment of AKI associated with NSAID type in TW. The finding that there was no significant difference in AKI risk between COX-2 selective and nonselective NSAIDs warrants particular attention. COX-2 inhibitors are often considered to carry a lower renal risk due to their reduced inhibition of COX-1–mediated prostaglandin synthesis in the kidney. However, TW-induced AKI may partly result from NSAIDs causing renal arteriolar constriction by inhibiting prostaglandin E2 and prostacyclin synthesis via COX inhibition [8], with COX-2 levels also enhanced in the kidney [12]. In addition, it was reported that in response to water deprivation, COX-2, but not COX-1, mRNA levels increase significantly in the renal medulla [43]. Therefore, the absence of a differential effect in our study may reflect these shared pathophysiological mechanisms. At the same time, it should be acknowledged that our study may not have had sufficient statistical power to detect modest differences between specific NSAID components, particularly in subgroup analyses. Additionally, potential exposure misclassification—such as differences in actual patient adherence or over-the-counter NSAID use—may have further attenuated true differences. These findings suggest that caution should be exercised when interpreting COX-2 inhibitors as safer alternatives within the context of triple therapy, and more targeted research is needed to confirm these results. Moreover, Lapi et al. reported that the AKI risk associated with TW may be highest in the early phase of treatment [4]. In our study, we focused on the 30-day period before AKI onset to capture the peak risk window and ensure a precise risk assessment. By evaluating NSAID use immediately before AKI onset, we minimized the influence of long-term exposure effects and provided a more accurate estimate of the short-term risk associated with TW. These results indicate that NSAID selection may not directly reduce the risk of AKI.

Our sensitivity analyses confirmed that the main findings of this study were robust under various conditions. When dialysis was included as the primary endpoint, the AKI incidence rate ratio for the TW group decreased compared to the two-drug group. This lower incidence ratio may reflect the fact that NSAIDs are less frequently prescribed to patients with advanced renal failure who are approaching dialysis initiation, thereby reducing the risk difference observed between the two groups. When AIN was included in the primary endpoint, the AKI incidence rate ratio for the TW group, compared to the two-drug group, remained similar (2.11 [95% CI: 1.72 − 2.58]). AIN is a rare but well-known consequence of NSAIDs use [44]. Approximately 70% of AIN cases are attributed to drug-induced causes, with antibiotics and proton pump inhibitors being the most commonly implicated agents rather than NSAIDs [45]. Therefore, the increase in outcomes observed when AIN was included may not fully reflect the adverse effects of NSAIDs but could be influenced by other medications prescribed concurrently. This suggests that the main analysis, which excluded AIN, was appropriate for assessing the NSAID-related AKI risk. Overall, these findings highlight that, while the specific definition of the primary outcome can influence the magnitude of the observed effects, the core result of increased AKI risk with TW remains robust. When the grace period was reduced or restricted to patients with at least two NSAID-dispensing events, the number of patients decreased, leading to some outcomes becoming non-significant. However, this did not overturn the results of the main analyses. The robustness of these findings within a short observation window suggests that NSAIDs may acutely increase the risk of AKI within a short period. This is consistent with our previous finding that the median time to AKI onset was 6.5 days [6]. These results emphasize the importance of monitoring short-term NSAID use, particularly in patients receiving RASIs and diuretics.

Nonetheless, this study has some limitations. First, the Japanese medical claims data used did not include laboratory values such as renal function, requiring AKI to be defined using ICD-10 codes. Although these codes have moderate sensitivity and high specificity [17], the severity of AKI and baseline renal function cannot be assessed. Therefore, we adjusted for the presence of chronic kidney disease. Second, the prescription and diagnostic data were recorded on the same day, making it unclear which event occurred first. Consequently, we excluded patients in whom AKI was diagnosed on the day TW was initiated, although some patients may have developed AKI shortly after receiving NSAIDs. Twenty patients were excluded from Analysis 1. Moreover, the case-crossover design is inherently susceptible to time-varying confounding, as acute clinical conditions (e.g., infections or trauma) may simultaneously trigger NSAID prescriptions and AKI onset. While we adjusted for surgical procedures and AKI-risk drugs, unmeasured transient factors may have introduced residual confounding. Moreover, the Poisson regression model used to estimate AKI incidence assumes a constant hazard over time. However, the risk of NSAID-induced AKI is likely to vary, particularly being higher shortly after drug initiation. This time-varying risk could have led to a misestimation of incidence rates under the constant hazard assumption. Nevertheless, our use of a complementary case-crossover analysis, which focuses on short-term exposure immediately prior to AKI onset, helps address this limitation by capturing time-dependent risk. Third, the actual NSAID intake could not be confirmed, as claims data reflect prescriptions but not adherence. As-needed NSAID prescriptions were excluded from the exposure definition because the actual timing and frequency of use could not be determined. Including them without confirmation of use could have led to exposure misclassification, while excluding them may have underestimated true NSAID exposure. Therefore, we conducted a sensitivity analysis that included only patients with at least two dispensing events, which did not overturn the main findings. Additionally, over-the-counter NSAID use was not captured, potentially leading to a misestimation of AKI risk in the two-drug group. Furthermore, whether dose compliance for RASIs and diuretics is maintained was unclear. Additionally, the relationship between drug intake and the risk of AKI remains unclear. In Analysis 1, diuretic use showed a dose-response trend with AKI incidence, warranting further investigation into its contribution to TW-induced AKI. Finally, although the used claims database includes individuals across various age groups, the majority of patients receiving the combination of RASIs, diuretics, and NSAIDs were older adults, resulting in a median age of 78 years in our cohort. This reflects the real-world prescribing patterns of TW therapy in Japan, rather than a structural bias of the database itself. Nevertheless, the age distribution limits the generalizability of our findings to younger populations, who were underrepresented in this study. Future studies that specifically target younger age groups are warranted to evaluate whether age modifies the risk of AKI under TW therapy.

## Conclusions

TW with the addition of NSAIDs in patients receiving RASIs and diuretics significantly increased the incidence of AKI in the period of TW, with an adjusted odds ratio that remained elevated after accounting for confounders. However, no significant risk differences were observed between NSAID components or COX-2 selectivity, suggesting that substituting one NSAID with another within TW does not mitigate AKI risk.

## Supplementary Information

Below is the link to the electronic supplementary material.

**Additional File 1**: **Supplemental Material 1**: Definitions of drugs and diseases used in the analysis, patient baseline characteristics and results of sensitivity analysis.

## Data Availability

The data that support the findings of this study are available from DeSC Healthcare Inc. but restrictions apply to the availability of these data, which were used under license for the current study, and so are not publicly available. Data are however available from the authors upon reasonable request and with permission of DeSC Healthcare Inc.

## Abbreviations

ACEI: Angiotensin-converting enzyme inhibitor
AIN: Acute interstitial nephritis
AKI: Acute kidney injury
aOR: Adjusted odds ratio
ARB: Angiotensin receptor blocker
ATC: Anatomical Therapeutic Chemistry
CI: Confidence interval
CKD: Chronic kidney disease
ICD-10: International Classification of Diseases: 10th Edition
ICU: Intensive care unit
NSAID: Non-steroidal anti-inflammatory drug
nsCOX-i: Nonselective cyclooxygenase inhibitor
RASI: Renin-angiotensin system inhibitor
sCOX2-i: Cyclooxygenase-2 selective inhibitor
TW: Triple Whammy
WHO: World Health Organization
