# Supplementary material for: NSAID-Induced acute kidney injury risk in patients on renin-angiotensin system inhibitors and diuretics: nationwide cohort study
Source: J Pharm Health Care Sci. 2025 Aug 18;11:77. doi: 10.1186/s40780-025-00485-8 (PMC12359997; doi:10.1186/s40780-025-00485-8)
Supplement: Supplementary file 1 — Additional File 1: Supplemental Material 1: Definitions of drugs and diseases used in the analysis, patient baseline characteristics and results of sensitivity analysis. [file 40780_2025_485_MOESM1_ESM.pdf]

Supplementary Materials

NSAID-Induced Acute Kidney Injury Risk in Patients on Renin-Angiotensin System Inhibitors and Diuretics: Nationwide Cohort Study

Authors: Yuki Kunitsu, Daiki Hira, Shunsaku Nakagawa, Masahiro Tsuda, Shin-ya Morita, Yosuke Yamamoto, and Tomohiro Terada

Supplemental Table 1. Definition of RASIs, diuretics, and NSAIDs

Supplemental Table 2. Definition of diseases based on ICD-10 diagnosis codes

Supplemental Table 3. Definition of drugs associated with AKI risk

Supplemental Table 4. The incidence rate of AKI by baseline characteristics in TW patients

Supplemental Table 5. Results of the sensitivity analysis in Analysis 1

Supplemental Table 6. Results of the sensitivity analysis in Analysis 2

Supplemental Figure 1. The design diagram of the RD cohort

Supplemental Table 1. Definition of RASIs, diuretics, and NSAIDs

Category	ATC code	Drug name
RASIs		
ACEIs	C09A	Alacepril, Benazepril hydrochloride, Captopril, Cilazapril hydrate, Delapril hydrochloride, Enalapril maleate, Imidapril hydrochloride, Lisinopril hydrate, Perindopril erbumin, Quinapril hydrochloride, Temocapril hydrochloride, Trandolapril
ARBs	C09C, C09D	Azilsartan, Candesartan cilexetil, Irbesartan, Losartan potassium, Olmesartan medoxomil, Sacbitol valsartan sodium hydrate, Telmisartan, Valsartan
Direct renin inhibitor	C09XA	Aliskiren fumarate
Diuretics		
Loop diuretics	C03CA	Azosemide, Bumetanide, Furosemide, Trasemide
Potassium-sparing diuretics	C03D	Eplerenone, Esaxelenone, Potassium kanrenoate, Spironolactone, Triamterene
Thiazide diuretics	C02LA01, C03AA, C09DA01–07, C09DX03	Benzylhydrochlorothiazide, Hydrochlorothiazide, Trichloromethiazide
Thiazide-like diuretics	C03BA	Indapamide, Meflucide, Methycran, Tripamide
Vasopressin V2 receptor antagonists	C03XA	Tolvaptan
NSAIDs		
nsCOX-i	M01A	Acemetacin, Aluminium flufenamate, Ampiroxicam, Diclofenac sodium, Flurbiprofen, Flurbiprofen axetil, Ibuprofen, Indomethacin, Indomethacin farnesyl, Ketoprofen, Loxoprofen sodium hydrate, Mefenamic acid, Moffezolac, Naproxen, Oxaprozin, Piroxicam, Planoprofen, Proglumetacin maleate, Surindac, Thiaprofenic acid
sCOX2-i	M01A	Celecoxib, Etodolac, Lornoxicam, Meloxicam, Nabumetone, Zartoprofen

Abbreviations: ACEIs, angiotensin-converting enzyme inhibitors. ARBs, angiotensin receptor blockers. ATC, Anatomical Therapeutic Chemistry. NSAIDs, non-steroidal anti-inflammatory drugs. nsCOX-I, nonselective cyclooxygenase inhibitors. RASIs, renin-angiotensin system inhibitors. sCOX2-i, cyclooxygenase-2 selective inhibitors.

Supplemental Table 2. Definition of diseases based on ICD-10 diagnosis codes

Diseases	ICD-10 code
Cancer	C00−C97
Diabetes mellitus	E10−E14
Heart failure	I110, I50
Hypertension	I10−I15
Ischemic heart disease	I20−I25
Acute kidney injury	N17
Chronic kidney disease	N18
Other renal disease	N00−N16, N19, N25−N29

Abbreviations: ICD-10, International Classification of Diseases, 10th Edition

Supplemental Table 3. Definition of drugs associated with AKI risk

Class name	ATC code
Antibiotics	A07A, J01, J02, J04, A02BD
Antineoplastics	L01
Antivirals	J05
Corticosteroids	H02A, H02B
Iodinated contrast agents	V08A

Abbreviations: AKI, acute kidney injury. ATC, Anatomical Therapeutic Chemistry.

Supplemental Table 4. The incidence rate of AKI by baseline characteristics in TW patients

Characteristics	n	AKI cases, n	Total follow-up duration, days	AKI incidence rate [95%CI] (/1,000 person-years)
All	41904	54	984848	20.0 [14.8−25.6]
Male	19857	28	409041	25.0 [16.1−34.8]
Age ≥75	30020	42	721914	21.2 [15.2−27.8]
Medical histories, n (%)				
Cancer	7656	16	163446	35.7 [20.1−53.6]
Diabetes mellitus	22006	29	511068	20.7 [13.6−28.6]
Heart failure	22266	34	511369	24.3 [16.4−32.8]
Hypertension	41623	54	980216	20.1 [14.9−25.7]
Ischemic heart disease	15160	24	343359	25.5 [15.9−36.1]
AKI[a]	85	0	2255	-
CKD	4873	12	100402	43.6 [21.8−69.1]
Other renal disease	9958	14	217806	23.5 [11.7−36.9]
Use of TW drugs, n (%)				
RASIs				
ACEIs	4983	9	105804	31.0 [13.8−51.7]
ARBs	37567	45	893824	18.4 [13.1−24.1]
Direct renin inhibitor	95	0	2927	-
Diuretics				
Loop diuretics	16300	31	368194	30.7 [20.8−41.6]

Potassium−sparing diuretics	8650	19	188804	36.7 [21.3−54.1]
Thiazide diuretics	19348	18	468431	14.0 [7.79−21.0]
Thiazide−like diuretics	4130	4	99701	14.6 [3.66−29.3]
Vasopressin V2 receptor antagonists	1227	6	21192	103.3 [34.4−189.5]
Kinds of diuretics used				
1	35034	34	840130	14.8 [10.0−20.0]
2	6025	16	128622	45.4 [25.5−68.1]
≥3	845	4	16096	90.7 [22.7−181.4]

[a]Patients who developed AKI within the past year were excluded.

AKI incidence rate: Calculated using the person-year method, dividing the number of AKI cases by the total follow-up period and presented per 1,000 person-years.

Abbreviations: ACEIs, angiotensin-converting enzyme inhibitors. AKI, acute kidney injury. ARBs, angiotensin receptor blockers. CI, confidence interval. CKD, Chronic kidney disease. NSAIDs, non-steroidal anti-inflammatory drugs. RASIs, renin-angiotensin system inhibitors. TW, Triple Whammy.

Supplemental Table 5. Results of the sensitivity analysis in Analysis 1

		Sensitivity Analysis				
	Main Analysis	AKI or dialysis as endpoint	AKI or AIN as endpoint	Grace period: 0 days	Grace period: 7 days	≥2 NSAIDs dispensings
Target patients, n	41904	41904	41904	16007	32979	16832
AKI cases, n	54	68	99	11	32	42
Total follow-up duration, days	984848	984848	983722	194117	533468	811793
AKI incidence rate [95%CI] (/1,000 person-years)	20.0 [14.8−25.6]	25.2 [19.3−31.5]	36.7 [29.7−44.2]	20.7 [9.40−33.8]	21.9 [14.4−30.1]	18.9 [1.3.5−24.7]
AKI incidence rate ratio (TW / RASIs and diuretics) [95%CI]	2.08 [1.58−2.74]	1.44 [1.13−1.84]	2.11 [1.72−2.58]	2.04 [1.12−3.71]	2.34 [1.64−3.34]	1.96 [1.44−2.67]

Abbreviations: AIN, acute interstitial nephritis. AKI, acute kidney injury. CI, confidence interval. NSAIDs, non-steroidal anti-inflammatory drugs. RASIs, renin-angiotensin system inhibitors. TW, Triple Whammy.

Supplemental Table 6. Results of the sensitivity analysis in Analysis 2

	Main Analysis	Sensitivity Analysis						
		AKI or dialysis as endpoint	AKI or AIN as endpoint	Grace period: 0 days	Grace period: 7 days	≥2 NSAIDs dispensings	Adjustment for ICU admission and calcineurin inhibitors use	Case period: 1–14 days pre-AKI Control periods: 31–44 & 61–74 days pre-AKI
Target patients (AKI cases), n (case period / control period)	2909/5818	4549/9098	5419/10838	741/1482	2003/6009	2909/5818	2909/5818	2909/5818
any NSAIDs use								
Users, n (case period / control period)	501/804	598/919	944/1,549	105/171	333/798	204/274	501/804	421/729
Adjusted[a] odds ratio [95%CI]	1.43 [1.16−1.77]	1.42 [1.18−1.71]	1.42 [1.18−1.71]	1.27 [0.81−2.00]	1.81 [1.30−2.52]	1.51 [1.17−1.95]	1.44 [1.16−1.77]	1.28 [1.01−1.63]
Type of NSAIDs use								
nsCOX-i use								
Users, n (case period / control period)	322/467	392/543	583/854	64/100	215/480	140/162	322/467	253/405

Adjusted[a] odds ratio [95%CI]	1.43 [1.13−1.80]	1.37 [1.12−1.68]	1.37 [1.12−1.68]	1.18 [0.71−1.95]	1.63 [1.14−2.35]	1.71 [1.26−2.35]	1.42 [1.13−1.79]	1.30 [0.99−1.70]
sCOX2-i use								
Users, n (case period / control period)	212/371	240/414	425/761	46/77	141/363	68/103	212/371	189/347
Adjusted[a] odds ratio [95%CI]	1.42 [1.01−2.00]	1.44 [1.05−1.96]	1.44 [1.05−1.96]	-	1.80 [1.05−3.10]	1.39 [0.93−2.07]	1.42 [1.01−2.00]	1.30 [0.88−1.94]
The relative adjusted[a] odds ratio of sCOX2-i to nsCOX-i	0.99 [0.66−1.50]	1.05 [0.72−1.52]	1.05 [0.72−1.52]	-	1.10 [0.58−2.10]	0.81 [0.49−1.34]	1.00 [0.66−1.51]	1.01 [0.62−1.62]
Component of NSAIDs use								
Diclofenac sodium hydrate use								
Users, n (case period / control period)	79/84	104/108	154/171	20/18	53/69	27/20	79/84	57/78
Adjusted[a] odds ratio [95%CI]	1.77 [1.17−2.70]	1.73 [1.20−2.49]	1.73 [1.20−2.49]	-	-	-	-	-
Loxoprofen sodium hydrate use								
Users, n (case period / control period)	228/350	268/401	410/636	45/79	154/369	97/123	228/350	184/315
Adjusted[a] odds ratio [95%CI]	1.42 [1.08−1.88]	1.32 [1.03−1.69]	1.32 [1.03−1.69]	-	1.51 [0.997−2.30]	1.63 [1.12−2.39]	1.42 [1.08−1.89]	1.13 [0.82−1.57]
Celecoxib use								

Users, n (case period / control period)	184/318	209/351	356/646	41/67	122/318	60/86	184/318	166/293
Adjusted[a] odds ratio [95%CI]	1.52 [1.05−2.21]	1.64 [1.16−2.31]	1.64 [1.16−2.31]	-	1.72 [0.96−3.10]	1.48 [0.97−2.28]	1.54 [1.06−2.23]	1.68 [1.09−2.59]

[a]adjustment factors: surgical procedures and the use of antibiotics, iodinated contrast agents, corticosteroids, antineoplastic agents, and antiviral drugs

Crude odds ratios were calculated only for items with an event count greater than 10. Adjusted odds ratios were calculated only for items with an event count greater than 10 times the number of covariates included.

Abbreviations: AIN, acute interstitial nephritis. AKI, acute kidney injury. CI, confidence interval. ICU, intensive care unit. NSAIDs, non-steroidal anti-inflammatory drugs. nsCOX-I, nonselective cyclooxygenase inhibitors. sCOX2-I, cyclooxygenase-2 selective inhibitors.

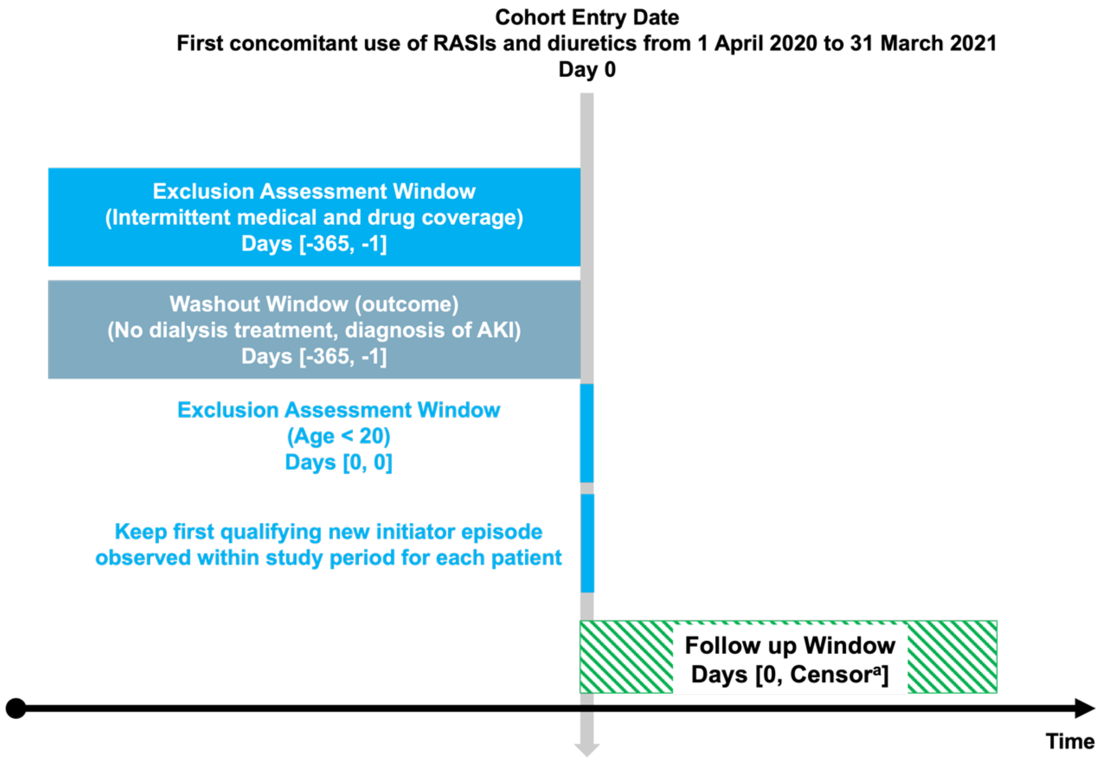

Supplemental Figure 1. The design diagram of the RD cohort

[a]Earliest of: Day 180, discontinuation of RASI or diuretics, disenrollment from database, end of the study period (March 31, 2022)

Abbreviations: AKI, acute kidney injury. RASIs, renin-angiotensin system inhibitors.
